# Quantifying Relative Diver Effects in Underwater Visual Censuses

**DOI:** 10.1371/journal.pone.0018965

**Published:** 2011-04-21

**Authors:** Luke C. Dickens, Christopher H. R. Goatley, Jennifer K. Tanner, David R. Bellwood

**Affiliations:** 1 Australian Research Council Centre of Excellence for Coral Reef Studies, James Cook University, Townsville, Australia; 2 School of Marine and Tropical Biology, James Cook University, Townsville, Australia; University of Glamorgan, United Kingdom

## Abstract

Diver-based Underwater Visual Censuses (UVCs), particularly transect-based surveys, are key tools in the study of coral reef fish ecology. These techniques, however, have inherent problems that make it difficult to collect accurate numerical data. One of these problems is the diver effect (defined as the reaction of fish to a diver). Although widely recognised, its effects have yet to be quantified and the extent of taxonomic variation remains to be determined. We therefore examined relative diver effects on a reef fish assemblage on the Great Barrier Reef. Using common UVC methods, the recorded abundance of seven reef fish groups were significantly affected by the ongoing presence of SCUBA divers. Overall, the diver effect resulted in a 52% decrease in the mean number of individuals recorded, with declines of up to 70% in individual families. Although the diver effect appears to be a significant problem, UVCs remain a useful approach for quantifying spatial and temporal variation in relative fish abundances, especially if using methods that minimise the exposure of fishes to divers. Fixed distance transects using tapes or lines deployed by a second diver (or GPS-calibrated timed swims) would appear to maximise fish counts and minimise diver effects.

## Introduction

SCUBA diving has greatly facilitated the collection and sampling of fishes on coral reefs. Underwater Visual Censuses (UVCs) are the most popular and practical method for studying the distribution and abundance of tropical reef fish populations [1]–[5]. Much of our understanding of marine ecosystems and the processes supporting them is founded on data collected via diver-based UVCs [2], [4]. Transects (i.e. belt transects) remain the most widely used non-destructive UVC technique [6]–[9]. However, in the quantitative study of coral reef fish communities, difficulties have been encountered when sampling populations and detecting spatio-temporal change [10]–[12]. Furthermore, inherent problems remain in the use of UVCs. Virtually every investigator evaluating these techniques has noted that all methods used to estimate fish density involve biases of some kind [4], [12]–[15]. Known biases can be accommodated or minimised; the greatest problem lies in dealing with biases of an unknown magnitude [4], [5].

One potentially serious but poorly understood problem in UVC sampling is the reaction of the fish to the diver: the diver effect [6], [14], [16]. SCUBA diving can be considered an invasive activity in regard to the distribution of fish [17]. The presence of a diver can cause some fishes to move away or hide, while others may be attracted, thereby decreasing or increasing counts [2], [16], [18]. Thus, it is possible that the diver effect may alter the results obtained from visual censuses, which can, in turn, lead to erroneous conclusions [3], [8], [12]. Negative associations with divers have been repeatedly documented and are a potential bias in all fish survey techniques, but this is especially seen in belt transects using tapes, leading to an underestimation of abundances and thus inferred functional impacts [9], [13], [19]–[22].

Despite all their problems, UVCs remain by far the most popular method available for surveying reef fish populations [2], [4]. In a review of 100 reef fish abundance studies over the last 10 years, 54% used tape-based belt transects, indicating that this is clearly the dominant UVC technique (see Figure S1 and Text S1). Further examination of these studies revealed that 69% do not acknowledge the diver effect. Of those that do, none have quantified the magnitude of the diver effect, though a few have used remedial measures to try and address this problem (e.g., [2], [6], [8], [23]). In these cases, it is generally assumed that the resumption of normal fish activity following disturbance by a diver is time-dependent [5], [19]. Many studies therefore use a 5 minute recovery period before starting a count [5], [24]–[26]. The efficacy of this approach is not known.

The interaction between sampling method and a species' behaviour was recognised over 30 years ago [18]. According to Kulbicki [2], of all the studies that had used UVCs, none had sufficiently examined the interaction between the observer and the fish, even when it was mentioned. While some previous studies have highlighted the potential influence of the diver effect, it has received comparatively little attention [2], [6], [8], [27]. Detailed studies on the effects of SCUBA divers on fish behaviour and distribution are rare and mostly anecdotal, largely due to the difficulty of measuring changes in fish behaviour in the field [2], [6], [8], [17], [28]. Therefore, the potential impact and bias of the diver effect remains unknown [2]–[4], [6]–[8], [21], [27]. As a result of the uncertainty surrounding the diver effect, considerable confusion exists in the literature; the reactions of fish to a diver's presence have been regarded as considerable by some authors and negligible by others [28].

Clearly, it is important to determine the magnitude of different sources of variation in abundance data and the sensitivity of measures to inherent biases in the methodology [11], [12], [21]. This is particularly important if temporal changes in fish population and community data are to be reliably estimated [12]. Although biases cannot be completely eliminated, the key is to recognise their potential effects on our understanding of the system and allow for them when drawing conclusions [4], [5]. Our goal, therefore, was to measure the relative diver effect in three different but commonly used fish census techniques, i.e. quantifying the extent to which diver presence may change the abundance of fishes recorded in each census technique. Specifically, the aims of the present study were to: (1) quantify the magnitude of relative diver effects in three UVC techniques and (2) measure among-family variation in the relative diver effect.

## Materials and Methods

### Ethics Statement

All activities are covered and approved by James Cook University Animal Ethics Review Committee (approval identification A1412). Only visual censuses of fish were conducted during this study; no animals were collected or manipulated.

All observations were undertaken during April and May 2009 at three sites along the reef crest of Pioneer Bay, located on the leeward side of Orpheus Island (18°35′S, 146°20′E), an inner-shelf island on the Great Barrier Reef (GBR), Australia. Orpheus Island, one of the Palm Islands, is a granitic continental island in the central section of the Great Barrier Reef Marine Park. Pioneer Bay has a well-developed fringing reef, typical of inner-shelf GBR reefs, with an extensive reef flat stretching approximately 150 m from the shoreline out to the reef crest (depth of 2–4 m) and down the reef slope to approximately 20 m [21], [29]. Orpheus Island has the largest marine reserve in the Palm Island group with the majority of the island's reef area zoned as a no-take area since 1987 [30]. Pioneer Bay is a protected Scientific Research Zone. All UVCs were conducted in this bay.

Our goal was to quantify the relative magnitude of the diver effect in three different census methods. Prior to censuses, locations along the reef crest with similar benthic configurations were identified within the three sites and marked with buoys. All censuses were conducted within three hours of high tide between 1000 and 1500 hrs to minimise confounding tide or time-of-day effects. To avoid localised disturbance the dive team entered the water at least 20 m from the site marker buoy, descended to the appropriate depth (2–3 m deep, 1 m above the substratum) and prepared for the survey (following [3]). A short fibreglass tape was used to estimate the 5 m width of the transect (2.5 m on either side of the diver's path) prior to censuses. At each buoyed location, three UVC techniques were executed.

As the majority of papers use tape transects (but with limited detail of how they were performed) we identified the three extreme cases in order to maximise differences in potential diver effects. The first was a 50 m fixed distance transect (while laying a 50 m fibreglass tape). Fishes were counted by an observer followed closely by a second diver laying the tape and stopping the observer after 50 m. This method was initially developed to minimise diver disturbance prior to counting [31]. In this fixed distance transect, fishes are just exposed to an initial disturbance.

Immediately after the fixed distance transect, fishes were re-censused along the 50 m tape to simulate the traditional practice of counting fishes after having laid a 50 m tape (the second diver following the observer to maintain the buddy pair). In this transect, fishes are exposed to the initial disturbance and the presence of the tape. Although recognised as a ‘diver effect’ it represents a complex response to ongoing diver presence and a ‘tape effect’ due to the presence of the tape. These two effects are invariably associated using this standard methodology.

Finally, fishes were counted again along the existing tape following a 5 minute acclimation period (as per [19], [32]). Again, the buddy followed the diver recording fish abundances. In total, 60 replicates were recorded for each UVC technique, 20 at each of the three sites (all censuses by LD). Seven key coral reef fish taxa (6 families) were included in the censuses: Acanthuridae, Chaetodontidae, wrasses (Labridae), Lutjanidae, parrotfishes (formerly Scaridae, now in the Labridae), Serranidae (Epinephelinae) and Siganidae. These taxa were selected as they represented the bulk of the visually apparent large-bodied benthic reef fishes in this location [33]. Only adult specimens with a total length greater than 10 cm were recorded, with the exception of the chaetodontids (typified by 7–11 cm individuals). Chaetodontids were included as they were easily identified, highly visible (even at a small size), and are a conspicuous family, often included in visual censuses.

In our analyses we compared the number of fishes recorded on each transect, i.e. the number of individuals per 250 m^2^. This metric was selected as it is the primary metric used in reef fish studies undertaking tape transects (in the literature survey over 90% of studies were recording fish densities, i.e. numbers per unit area). It is also the most likely to be responsive to diver effects (rather than species richness). In the analyses, we first compared the fixed distance and immediate censuses. As they were covering the same area, the samples were non-independent and densities were therefore examined using paired t-tests on total fish densities and densities within each of the focal taxa. To investigate the extent to which a 5 minute acclimation period would reduce the diver effect, the initial count was also compared to the final count (after the tape had been swum over twice). Prior to analyses, all data were log transformed (log_10_ n +1) to ensure that homogeneity of variance and normality were within acceptable limits. In all t-tests, a Bonferroni correction was used, resulting in an adjusted alpha-level (i.e. α = 0.05/8 = 0.006).To examine the variation in the abundance of individual species in the three separate censuses (fixed distance, immediate and after 5-minutes), mean species' abundances were examined using a principal component analysis (PCA; covariance analysis of log_10_ n +1 transformed data).

## Results

**Figure 1:**
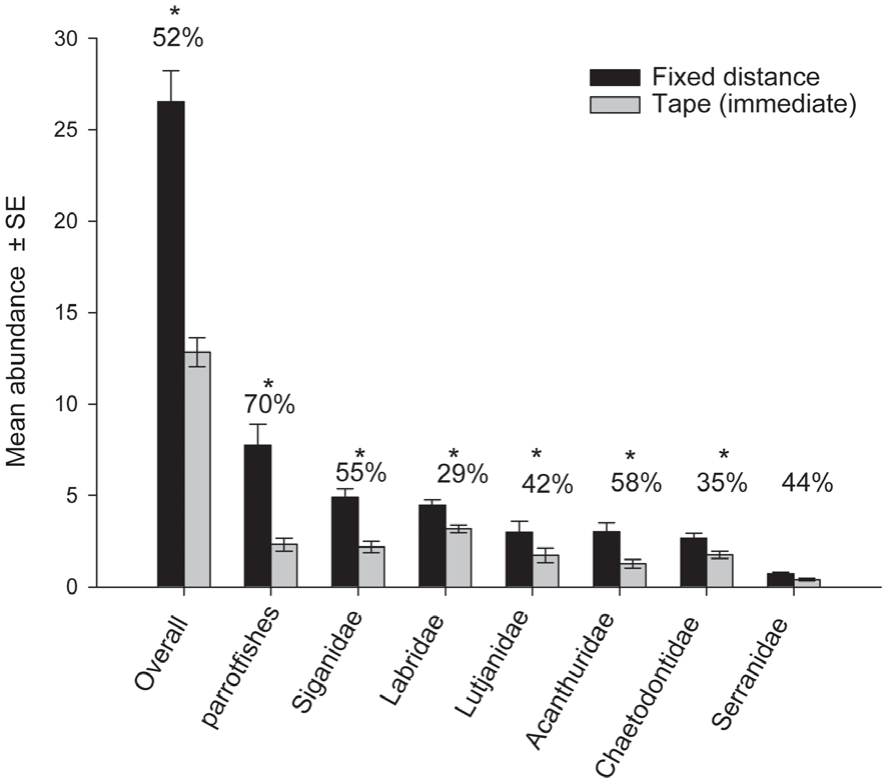
Relative diver effects – fixed distance and immediate return. Relative diver effects on estimated reef fish densities comparing counts over a fixed distance 50 m transect and counts along the tape immediately after deployment. Values indicate the proportional decrease in abundance; asterisks represent significant differences using a Bonferroni corrected alpha-value (*p*<0.006). The parrotfishes formerly in the family Scaridae are now a distinct lineage within in Labridae [56].

In terms of all taxa combined, the immediate return tape transect yielded less than half the number of fishes recorded using the initial fixed distance transect, with only 12.8±0.8 (mean ± SE) fishes compared to 26.5±1.7, respectively (Figure 1). When compared with the fixed distance transect, this represents a loss of 52%. A similar pattern was observed when each family or group was examined separately (all *p*<0.05), with all families except the Serranidae showing statistical significance after Bonferroni adjusted *p*-values were used (Table 1).

**Table 1:** Paired t-tests – fixed distance and immediate return.

	*t*	*df*	*P*
Overall fish abundance	12.495	59	**<0.001**
Acanthuridae	3.948	59	**<0.001**
Chaetodontidae	2.964	59	**<0.001**
Labridae	3.540	59	**<0.001**
Lutjanidae	3.178	59	**<0.001**
parrotfishes	9.179	59	**<0.001**
Serranidae	2.041	59	0.046
Siganidae	5.766	59	**<0.001**

Paired t-tests showing differences for overall fish abundance and abundances within each family between fixed distance and immediate return tape transects. P-values marked in bold show significance (using Bonferroni adjusted alpha levels α<0.006).

**Figure 2:**
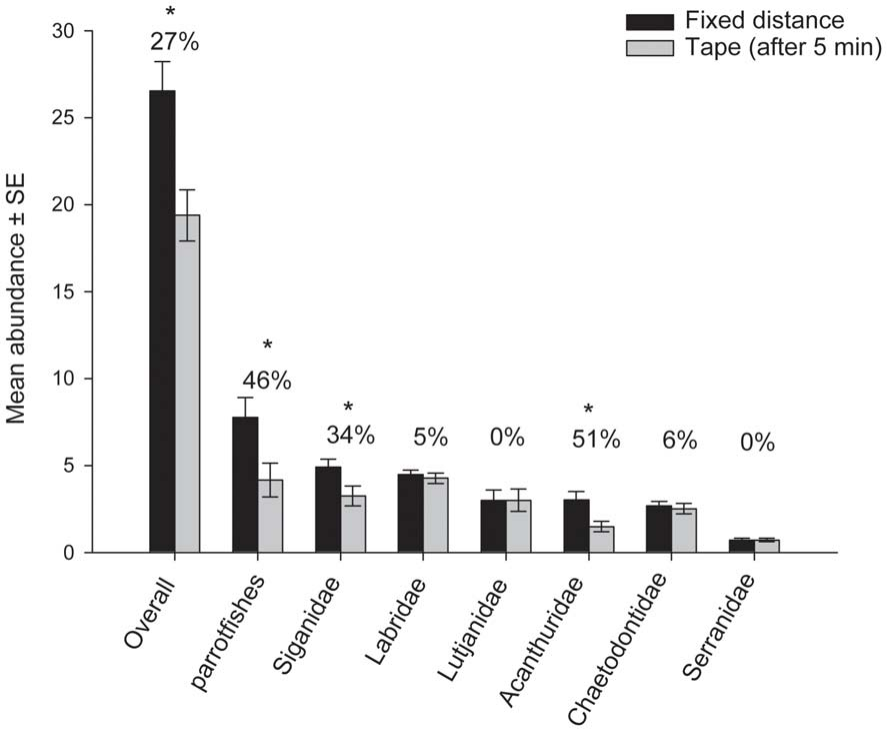
Relative diver effects – fixed distance and 5 minute waiting period. Relative diver effects on estimated reef fish densities comparing counts over a fixed distance 50 m transect and counts after a 5 minute waiting period. Values indicate the proportional decrease in abundance, asterisks represent significant differences using a Bonferroni corrected alpha-value (*p*<0.006).

Despite the rather large declines reported for the immediate transects, numbers did recover to some extent after a 5 minute waiting period. Comparing the initial fixed distance counts with those after the 5 minute waiting period resulted in a difference in all fishes combined of just 27% (Figure 2). This difference was significant for overall fish abundance even with Bonferroni correction (Table 2) and was supported by an analysis to compare all three 50 m transects simultaneously (a repeated measures ANOVA, because of non-independence, see Tables S1, S2, and S3). Analyses of individual taxa after 5 minutes found that counts were generally lower than the initial counts (Figure 2), however, this was statistically significant only for the parrotfishes, Siganidae and Acanthuridae (Table 2), compared to six of the seven groups without a 5 min waiting period.

**Table 2:** Paired t-tests – fixed distance and 5 minute waiting period.

	*t*	*df*	*P*
Overall fish abundance	5.110	59	**<0.001**
Acanthuridae	3.666	59	**<0.001**
Chaetodontidae	0.717	59	0.476
Labridae	0.707	59	0.482
Lutjanidae	0.721	59	0.474
parrotfishes	5.548	59	**<0.001**
Serranidae	0.036	59	0.971
Siganidae	3.255	59	**<0.001**

Paired t-tests showing differences for overall fish abundance and within each family between fixed distance and tape (after 5 minutes) transects. P-values marked in bold show significance (using Bonferroni adjusted alpha levels α<0.006).

**Figure 3:**
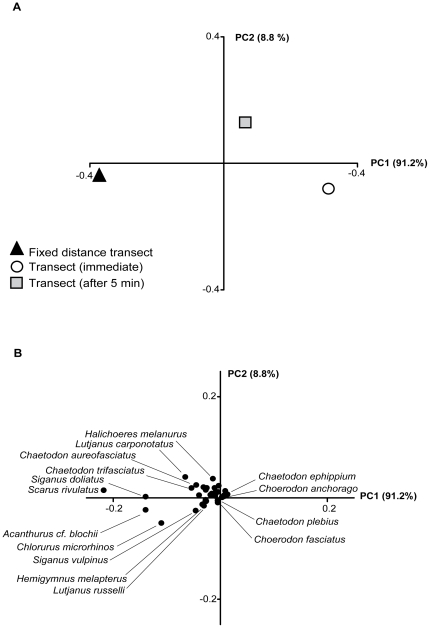
Principal components analysis showing species level differences in mean fish abundance between the three UVC techniques. (**A**) All 3 transects are clearly separated along PC1. (**B**) Vector plot showing the fish species driving patterns in relation to the three transects shown in (**A**).

The PCA (Figure 3) revealed extremely high scores on PC1 (91.2%) indicating that almost all variation could be explained by the presence or absence of species lying along this axis (PC2 explained just 8.8%). PC1 clearly separates the initial fixed distance and immediate transects, with the 5 min transect lying in line but closest to the immediate transect. High scoring species vectors were invariably sited towards the initial fixed distance transect. This indicates that the difference between the transects was due to the presence or absence of species rather than a replacement of one species with another. It appears that all responsive species were missing in the immediate transect. Following the family level analyses above, the most diver negative species were the parrotfishes *Scarus rivulatus* and *Chlorurus microrhinos*, the surgeonfish *Acanthurus* cf. *blochii* and the siganids *Siganus doliatus* and *S*. *vulpinus*.

## Discussion

We recorded a marked decline in fish abundance as a result of ongoing diver presence. Overall, there was a 52% decrease in the mean number of fish recorded between fixed distance and immediate tape transects. The maximum recorded decrease was 70% for parrotfishes. A comparable diver effect was found, to varying extents, in all reef fish groups examined.

The tendency of fish to avoid divers is supported by the literature in terms of both the magnitude and nature of the diver effect [9], [17], [34], [35]. Stanley and Wilson [23], for example, found that the presence of SCUBA divers conducting visual point surveys resulted in a decline in the mean density of fishes between 41 and 77% (with a mean reduction of 60%). This compares closely with the 29–70% (overall mean reduction of 52%) mean decreases recorded herein. Similar negative reactions have been reported in both freshwater and enclosed saltwater systems, in which the presence of a SCUBA diver resulted in lower fish abundances being recorded [16], [25]. These negative reactions are also supported by the observations of Kulbicki [2] and Feary et al. [36] that suggest that diver presence can adversely affect fish behaviour.

The findings of the present study, however, contrast with those of Watson and Harvey [28], who found several fish species within a marine protected area (MPA) to be attracted to divers (see also [9], [17]). Pioneer Bay is also protected and has a long history of exposure to SCUBA divers [29], yet a negative diver effect was still evident. We recorded no positive diver responses in any species at these sites during this study, although positive responses to divers have been observed in some labrid taxa (*Choerodon*, *Thalassoma*) in neighbouring bays when clove oil is in use (DRB pers. obs.). Overall, although diver effects are complex with negative and positive behaviour recorded in the literature, the magnitude of diver-mediated effects found on coral reefs in the present study is comparable, in both nature and magnitude, to a number of previous studies in other aquatic habitats.

The physiological basis for the negative reaction is not known. Of all stimuli, sound is most frequently identified in diver avoidance [8], [27], [37], [38]. However, sound is unlikely to account for the observed changes in fish abundance. Much of the sound produced by SCUBA is at low frequencies, where fish hearing is most sensitive [27], [38]. This noise is estimated to be detectable by most fish species at distances of at least 200 m [27]. However, if sound was the main stimulus, we would expect any affected fish to leave the area prior to their visual detection when laying the tape, and for the relative diver effect to be limited. Indeed, there is considerable evidence at this study site of fishes avoiding divers before they can be seen. Both siganids and batfish have been documented to be present in the area, based on remote underwater video, yet remain undetected using UVC methods [29], [39]. It appears that these fishes leave the area long before divers arrive, presumably in response to sound. We are therefore looking at the response of fishes that remain despite the presence of diver-related sound. For these fishes, other stimuli must be involved.

Our results suggest that vision is the main stimulus for the fishes encountered on the transects. It appears that the ongoing visible presence of divers exerts a negative diver effect and is largely responsible for the strong patterns seen in the present study. This may be expected given the importance of the visual system for coral reef fishes [38], [40], and a diver could be interpreted by fish as a predator [17], [28]. Regardless of the reasons, the ongoing visible presence of the diver appears to account for the initial decline in the immediate transects (and the variable response to remedial measures).

Although the response to the diver appears to be the primary stimulus it must be noted that there is also a possible ‘tape effect’. These two issues are inextricably associated in the standard methodology, as fishes are exposed to the ongoing presence of both the diver and tape. Although the tape effect appears to be relatively small, some fishes have been observed to respond negatively to the tapes, approaching them and then changing swimming direction or fleeing. A comparable pattern was seen with stationary video quadrats which are now removed before filming [29]. As the goal of this study was to establish the magnitude of the diver effect (*sensu lato* including the tape effect) in a traditional approach the two factors are combined. However, it may be useful in future to separate the relative impact of the diver(s) and tape.

One additional limitation of the present study is that only those species that remain visible can be recorded and considered in terms of diver-based reactions [3], [33], [41]. Some reef species have only been observed when a diver was absent, generally using remote video cameras [29], [39]. The diver effects recorded in the present study, therefore, are all relative and represent the increase in disturbance as a result of tape deployment and persistent diver presence. They may more accurately be regarded as a ‘tape and ongoing diver presence’ effect on visually apparent taxa.

The relative diver effect was evident to different extents among the seven groups examined. This was statistically significant in six of the seven groups with mean decreases of 29–70% from initial abundances. The herbivorous parrotfishes, Acanthuridae and Siganidae [42] were abundant in initial censuses, but were greatly affected by the ongoing presence of divers in return tape transects. Parrotfishes are widely documented to be skittish and highly mobile, thus having a reduced likelihood of detection, often moving away from approaching divers in the area [5], [21], [28], [29], [36], [39], [43], [44]. The locally abundant and functionally important species *Scarus rivulatus* and *Chlorurus microrhinos* [29], [45] were particularly sensitive to ongoing diver presence. The acanthurids are regarded as less mobile than the parrotfishes [5] although some species are acknowledged to be wary [46], possibly explaining why they are the next most diver-affected taxon. Siganids (primarily *Siganus doliatus*) were the least responsive of the herbivores. There is a possibility than rather than being wary, some fishes may have been initially attracted to the divers then lost interest. This behaviour is certainly possible in marine protected areas if fishes have been fed [2], [28]. However, this diver positive behaviour is highly unlikely at this site as there is no fish feeding in this area. Furthermore, no diver positive behaviour has previously been observed by the authors at this site despite over 30 years working in the area. In contrast, as noted at this site [29], [39] and in other locations [5], [28], [36], [43], [44], strong diver negative responses are regularly reported, especially in parrotfishes.

The Chaetodontidae and wrasses were two of the least-affected groups. Chaetodontids are often closely associated with the structure of the benthic reef habitat [47] and may have restricted home ranges [46], limiting their flight response. Likewise, some wrasses have limited home ranges and may be attracted to divers [46]. A similar pattern is seen in the two remaining families, Serranidae and Lutjanidae, which displayed a decrease in abundance in the immediate return tape transect. However, this was not statistically significant in the Serranidae, possibly reflecting small sample sizes. The five minute wait did appear to be an effective remedial action, especially for these non-herbivorous families. Given that the tapes had been swum over twice, a standard 5 minutes would appear to help reduce diver effects for these families.

The diver-negative reactions recorded in the present study suggest that absolute numbers, and therefore the functional impact of mobile reef fishes, may be underestimated when using standard UVC techniques. In all 7 families, the immediate return census was lower than the fixed distance count despite the fact that Pioneer Bay has been exposed to active SCUBA-based research for more than 30 years [29]. Given the magnitude of negative diver effects found in the present study, one might ask how the results would differ if the fishes were targets for fishing. The behaviour of species targeted by spear-fishing in the presence of divers is documented to be mostly negative, as one would expect, with 70–80% of fish exhibiting an escape response [48]. However, the diver effect is believed to extend far beyond targeted species. Where spear-fishing is intense, both target and non-target fish are reported to avoid humans. Fishes in marine reserves are less wary of divers and more likely to be detected than fishes in fished areas [2], [5], [36], [49], [50]. In these fished areas, the magnitude of the relative diver effect is likely to be greater than in protected areas.

In the present study, the parrotfishes (a non-harvested species in a marine protected no-take area) exhibited a 70% decrease in mean abundance (comparing the fixed distance transect with the immediate return tape transect). Such variation calls for caution when comparing studies using different methods. The magnitude of change and the potential for further fishing-mediated variation in absolute and relative densities are also of concern. The magnitude of the diver effect recorded herein approximates the differences described in previous studies of parrotfish densities in the literature related to MPAs (e.g., [50]), cross-shelf variation (e.g., [51], [52]), and fishing pressure (e.g., [53]). Furthermore, taxonomic variability in the diver effect may be particularly important in studies where variation in taxonomic or functional composition is examined in relation to spatial or temporal variation in fishing pressure. The presence of marine parks may increase the attraction of fishes to divers (especially if the fish are fed), while open (fished) areas are likely to increase diver-negative responses. For example, recent work has recorded significant increases in flight distances of fishes outside customary marine closure areas, especially in parrotfishes [36].

Our results suggest that the censusing of fishes after laying a measuring tape can have a profound effect on fish counts. While this may not be detrimental for analyses of relative abundances within a single study, it may severely limit our ability to combine visual census data in meta-analyses, or to compare values among studies. It appears that the most robust and accurate visual censuses will be based on active recording, as in fixed distance transects (where the observer or a second diver deploys a line [31]) or timed swim transects (with distances calculated from GPS readings [54]), where both the diver and tape effects are minimised.

Recent publications have identified significant problems with tape-based transects. This includes detectability, where small or cryptic fishes are only effectively recorded close to the observer, and transect length effects, where there is an anomalous peak in counts at the start and end of transects [24], [55]. From this published evidence, and the results herein, a clear picture is emerging. Accuracy of UVCs may be enhanced by: (1) Minimising problems detecting fishes [55], perhaps by stratified sampling, where large fishes are sampled in large transects while smaller specimens are sampled simultaneously by a second diver using smaller transects [54]. (2) Minimising start and end anomalies [24], by using long transects. And, (3) using methods that record fishes at the first encounter. This minimises any diver effect, eliminates any tape effect, and saves time implementing remedial measures such as a waiting time. Thus fixed distance transects with the distance measured by the observer or a second diver, GPS-calibrated timed swims, or permanent (unobtrusive) transects would appear to be more accurate than traditional point counts or standard tape transects. Overall, for large visually-detectable fishes, long, first encounter, size-stratified censuses appear to be the best currently available UVC methods.

Fortunately, as most studies are interested in spatial or temporal changes in the relative abundance of fishes (rather than absolute numbers), diver effects, if constant, will have only a limited effect on interpretations using existing data. Thus, while the diver effect cannot be completely eliminated from UVCs, awareness of its potential direction and magnitude will hopefully permit a better understanding of the abilities and limitations of UVCs.

## Supporting Information

Figure S1 **Relative frequency of Underwater Visual Census techniques used to survey abundance of coral reef fishes.** Studies (n =  100) were collated using Web of Science with the search terms “abundance” and “reef fish” published 1999–2009. To avoid bias caused by authors favouring particular techniques, primary authors were only used once. For studies using multiple techniques, publications were included in more than one category. The category “other” incorporated studies using manta tow, distance sampling, sonar or video.
(TIF)

Table S1 **Repeated measures ANOVA for overall fish abundance showing no site effect.** There is a significant difference (marked in bold) between the three UVC techniques (fixed distance, tape (immediate return) and tape (after 5 min).
(DOC)

Table S2 **Tukey's HSD post-hoc test showing significance between different UVC techniques.** All 3 transects (fixed distance, tape (immediate return) and tape (after 5 minutes)) differ significantly from one another (values marked in bold).
(DOC)

Table S3 **One-way ANOVA for overall fish abundance and UVC techniques.** Significant values are marked in bold.
(DOC)

Text S1 Sources used in literature evaluation.
(DOC)
