# Supplementary figures and images for: Quantifying Relative Diver Effects in Underwater Visual Censuses
Source: PLoS One. 2011 Apr 21;6(4):e18965. doi: 10.1371/journal.pone.0018965 (PMC3080881; doi:10.1371/journal.pone.0018965)

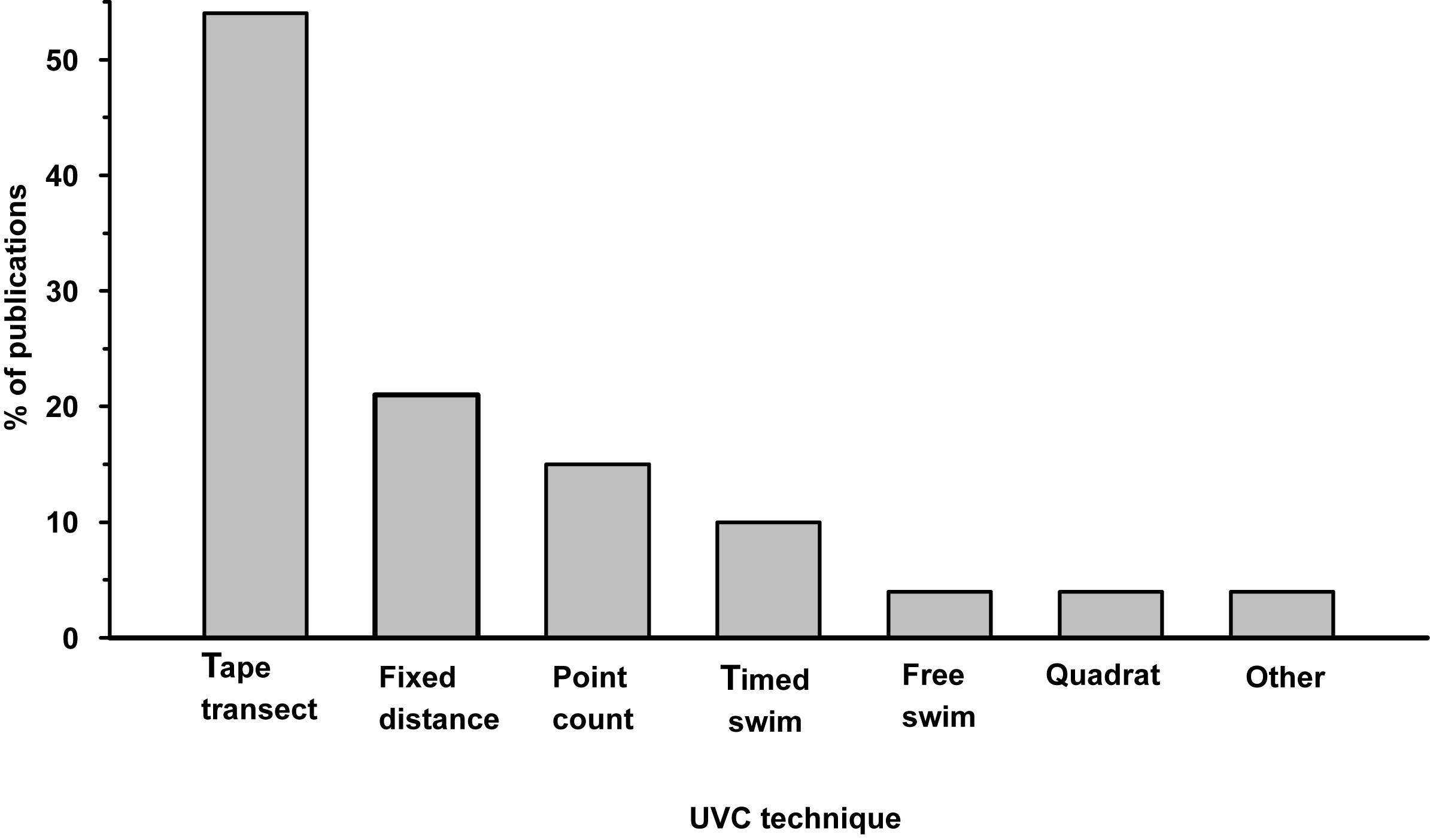

Supplement: Figure S1 — Relative frequency of Underwater Visual Census techniques used to survey abundance of coral reef fishes. Studies (n = 100) were collated using Web of Science with the search terms “abundance” and “reef fish” published 1999–2009. To avoid bias caused by authors favouring particular techniques, primary authors were only used once. For studies using multiple techniques, publications were included in more than one category. The category “other” incorporated studies using manta tow, distance sampling, sonar or video. (TIF) [file pone.0018965.s001.tif]
