# Supplementary material for: Quantifying Relative Diver Effects in Underwater Visual Censuses
Source: PLoS One. 2011 Apr 21;6(4):e18965. doi: 10.1371/journal.pone.0018965 (PMC3080881; doi:10.1371/journal.pone.0018965)
Supplement: Table S1 — Repeated measures ANOVA for overall fish abundance showing no site effect. There is a significant difference (marked in bold) between the three UVC techniques (fixed distance, tape (immediate return) and tape (after 5 min). (DOC) [file pone.0018965.s002.doc]

**Table S1**

| Source of variation | SS | *df* |  | MS | *F* | *P* |
| --- | --- | --- | --- | --- | --- | --- |
| Site | 0.055 | 2 |  | 0.028 | 0.061 | 0.940 |
| UVC Techniques | 13.980 | 2 |  | 6.990 | 56.931 | **<0.001** |
| Site*UVC Techniques | 0.321 | 4 |  | 0.080 | 0.653 | 0.626 |
| Error | 13.997 | 114 |  | 0.123 |  |  |
