# Supplementary material for: Quantifying Relative Diver Effects in Underwater Visual Censuses
Source: PLoS One. 2011 Apr 21;6(4):e18965. doi: 10.1371/journal.pone.0018965 (PMC3080881; doi:10.1371/journal.pone.0018965)
Supplement: Table S2 — Tukey's HSD post-hoc test showing significance between different UVC techniques. All 3 transects (fixed distance, tape (immediate return) and tape (after 5 minutes)) differ significantly from one another (values marked in bold). (DOC) [file pone.0018965.s003.doc]

**Table S2**

| **UVC Technique** | **Fixed distance** | **Tape (immediate)** | **Tape (after 5 min)** |
| --- | --- | --- | --- |
| Fixed distance |  | **<0.0001** | **<0.0001** |
| Tape (immediate) | **<0.0001** |  | **<0.0001** |
| Tape (after 5 min) | **<0.0001** | **<0.0001** |  |
