# Supplementary material for: Quantifying Relative Diver Effects in Underwater Visual Censuses
Source: PLoS One. 2011 Apr 21;6(4):e18965. doi: 10.1371/journal.pone.0018965 (PMC3080881; doi:10.1371/journal.pone.0018965)
Supplement: Table S3 — One-way ANOVA for overall fish abundance and UVC techniques. Significant values are marked in bold. (DOC) [file pone.0018965.s004.doc]

**Table S3**

| Source of variation | SS | *df* |  | MS | *F* | *P* |
| --- | --- | --- | --- | --- | --- | --- |
| UVC Techniques | 3.034 | 2 |  | 1.517 | 30.555 | **<0.001** |
| Error | 8.789 | 177 |  | 0.050 |  |  |
