# Supplementary material for: Quantifying Relative Diver Effects in Underwater Visual Censuses
Source: PLoS One. 2011 Apr 21;6(4):e18965. doi: 10.1371/journal.pone.0018965 (PMC3080881; doi:10.1371/journal.pone.0018965)
Supplement: Text S1 — Sources used in literature evaluation. (DOC) [file pone.0018965.s005.doc]

**Supporting Information**

**Text S1**

**Sources used in literature evaluation:**

Acosta CA, Robertson DN (2002) Diversity in coral reef fish communities: the effects of habitat patchiness revisited. Mar Ecol Prog Ser 227:87-96

Adams AJ, Ebersole JP (2002) Use of back-reef and lagoon habitats by coral reef fishes. Mar Ecol Prog Ser 228:213-226

Adjeroud M, Augustin D, Galzin R, Salvat B (2002) Natural disturbances and interannual variability of coral reef communities on the outer slope of Tiahura (Moorea, French Polynesia): 1991 to 1997. Mar Ecol Prog Ser 237:121-131

Arias-Gonzalez JA, Legendre P, Rodriguez-Zaragoza FA (2008) Scaling up beta diversity on Caribbean coral reefs. J Exp Mar Biol Ecol 366:28-36

Arnal C, Kulbicki M, Harmelin-Vivien M, Galzin R, Morand S (2002) Patterns of local distribution of *Labroides dimidiatus* in French Polynesian atolls. Environ Biol Fish 63:9-15

Ashworth JS, Ormond RFG (2005) Effects of fishing pressure and trophic group on abundance and spillover across boundaries of a no-take zone. Biol Conserv 121:333-344

Ault JS, Smith SG, Bohnsack JA, Luo J, Harper DE, McClellan DB (2006) Building sustainable fisheries in Florida’s coral reef ecosystem: positive signs in the Dry Tortugas. Bull Mar Sci 78:633-654

Bean K, Jones GP, Caley MJ (2002) Relationships among distribution, abundance and microhabitat specialisation in a guild of coral reef triggerfish (family Balistidae). Mar Ecol Prog Ser 233:263-272

Bellwood DR, Wainwright PC (2001) Locomotion in labrid fishes: implications for habitat use and cross-shelf biogeography on the Great Barrier Reef. Coral Reefs 20:139-150

Benfield S, Baxter L, Guzman HM, Mair JM (2008) A comparison of coral reef and coral community fish assemblages in pacific panama and environmental factors governing their structure. J Mar Biol Assoc UK 88:1331-1341

Bonin MC, Srinivasan M, Almany GR, Jones GP (2009) Interactive effects of nterspecific competition and microhabitat on early post-settlement survival in a coral reef fish. Coral Reefs 29:265-274

Booth DJ, Beretta GA (2002) Changes in a fish assemblage after a coral bleaching event. Mar Ecol Prog Ser 245:205-212

Bozec Y-M, Kulbicki M, Chassot E, Gascuel D Trophic signature of coral reef fish assemblages: Towards a potential indicator of ecosystem disturbance. Aquat Living Recour 18:103-109

Brokovich E, Einbinder S, Shashar N, Kiflawi M, Kark S (2008) Descending into the twilight-zone: changes in coral reef fishassemblages along a depth gradient down to 65 m. Mar Ecol Prog Ser 371:253-262

Camargo C, Maldonado JH, Alvarado E, Moreno-Sanchez R, Mendoza S, Manrique N, Mogollon A, Osorio JD, Grajales A, Sanchez JA (2009) Community involvement in management for maintaining coral reef resilience and biodiversity in southern Caribbean marine protected areas. Biodivers Conserv 18:935-956

Campbell SJ, Pardede ST (2006) Reef fish structure and cascading effects in response to artisanal fishing pressure. Fish Res 79:75-83

Caselle JE (1999) Early post-settlement mortality in a coral reef fish and its effect on local population size. Ecol Monogr 69:177-194

Ceccarelli DM, Jones GP, McCook LJ (2005) Foragers versus farmers: contrasting effects of two behavioural groups of herbivores on coral reefs. Oecologia 145:445-453

Chabanet P (2002) Coral reef fish communities of Mayotte (western Indian Ocean) two years after the impact of the 1998 bleaching event. Mar Freshwater Res 53:107-113

Chapman MR, Kramer DL (1999) Gradients in coral reef fish density and size across the Barbados Marine Reserve boundary: effects of reserve protection and habitat characteristics. Mar Ecol Prog Ser 181:81-96

Cheal AJ, Coleman G, Delean S, Miller I, Osborne K, Sweatman H (2002) Responses of coral and fish assemblages to a severe but short-lived tropical cyclone on the Great Barrier Reef, Australia. Coral Reefs 21:131-142

Chittaro PM (2004) Fish-habitat associations across multiple spatial scales. Coral Reefs 23:235-244

Dahlgren CP, Eggleston DB (2001) Spatio-temporal variability in abundance, size and microhabitat associations of early juvenile Nassau grouper *Epinephelus striatus* in an off-reef nursery system. Mar Ecol Prog Ser 217:145-156

DeMartini EE, Friedlander AM, Sandin SA, Enric S (2008) Differences in fish-assmblage structure between fished and unfished atolls in the northern Line Islands, central Pacific. Mar Ecol Prog Ser 365:199-215

Dulvy NK, Polunin NVC, Mill AC, Graham NAJ (2004) Size structural change in lightly exploited cora reef fish communities: evidence for weak indirect effects. Can J Fish Aquat Sci 61:466-475

Edgar GJ, Barrett NS, Morton AJ (2004) Biases associated wit the use of underwater visual census techniques to quantify the density and size-structure of fish populations. J Exp Mar Biol Ecol 308:269-290

Eggleston DB, Dahlgren CP, Johnson EG (2004) Fish density, diversity and size-structure within multiple back reef habitats of Key West National Wildlife Refuge. Bull Mar Sci 75:175-204

Emslie MJ, Cheal AJ, Sweatman H, Delean S (2008) Recovery from disturbance of coral and reef fish communities on the Great Barrier Reef, Australia Mar Ecol Prog Ser 371:177-190

Evans RD, Russ GR, Kritzer JP (2008) Batch fecundity of *Lutjanus carponotatus* (Lutjanidae) and implications of no-take marine reserves on the Great Barrier Reef, Australia Coral Reefs 27:179-189

Ferreira CEL, Goncalves JEA (2006) Community structure and diet of roving herbivorous reef fishes in the Abrolhos Archipelago, south-western Atlantic J Fish Biol 69:1533-1551

Floeter SR, Krohling W, Gasparini JL, Ferreira CEL, Zalmon IR (2007) Reef fish community structure on coastal islands of the southeastern Brazil: the influence of exposure and benthic cover. Environ Biol Fish 78:147-160

Fox RJ, Bellwood, DR (2007) Quantifying herbivory across a coral reef depth gradient. Mar Ecol Prog Ser 339:49-59

Francini-Filho RB, Moura RL (2008) Evidence for spillover of reef fishes from a no-take marine reserve: An evaluation using the before-after control-impact (BACI) approach. Fish Res 93:346-356

Friedlander AM, Brown EK, Jokiel PL, Amith WR, Rodgers KS (2003) Effects of habitat, wave exposure, and maire protected area status on coral reef fish assemblages in the Hawaiian archipelago. Coral Reefs 22:291-305

Fulton CJ, Bellwood, DR (2004) Wave exposure, swimming performance, and the structure of tropical and temperate reef fish assemblages. Mar Biol 144:429-437

Galal N, Ormond RFG, Hassan O (2002) Effect of a network of no-take reserves in increasing catch per unit effort and stocks of exploited reef fish at Nabq, South Sinai, Egypt. Mar Freshwater Res 53:199-205

Garpe KC, Ohman MC (2007) Non-random habitat use by coral reef fish recruits in Mafia Island Marine Park, Tanzania. Afr J Mar Sci 29:187-199

Graham NAJ, Dulvy NK, Jennings S, Polunin NVC (2005) Size-spectra as indicators of the effects of fishing on coral reef fish assemblages. Coral Reefs 24:118-124

Gratwicke B, Speight MR (2005) The relationship between fish species richness, abundance and habitat complexity in a range of shallow tropical marine habitats. J Fish Biol 66:650-667

Grober-Dunsmore R, FrazerTK, Lindberg WJ, Beets J (2007) Reef fish and habitat relationships in a Caribbean seascape: the importance of reef context. Coral Reefs 26:201-216

Gust N, Choat JH, McCormick MI (2001) Spatial variability in reef fish distribution, abundance, size and biomass: a multi-scale analysis. Mar Ecol Prog Ser 214:237-251

Halford A, Cheal AJ, Ryan D, Williams DMcB (2004) Resilience to large-scale disturbance in coral and fish assemblages on the Great Barrier Reef. Ecology 85:1892-1905

Hamilton SL, White JW, Caselle JE, Swearer SE, Warner RR (2006) Consistent long-term spatial gradients in replenishment for an islad population of a coral reef fish. Mar Ecol Prog Ser 306:247-256

Harborne AR, Mumby PJ, Kappel CV, Dahlgren CP, Micheli F, Holmes KE, Sanchirico JN, Broad K, Elliot IA, Brumbaugh DR (2008) Reserve effects and natural variation in coral reef communities. J Appl Ecol 45:1010-1018

Hawkins JP, Roberts CM, Gell FR, Dytham C (2007) Effects of trap fishing on reef fish communities. Aquatic Conserv: Mar Freshw Ecosyst 17:111-132

Hoey AS, Bellwood DR (2008) Cross-shelf variation in the role of parrotfishes on the Great Barrier Reef. Coral Reefs 27:37-47

Holbrook SJ, Schmitt RJ, Brooks AJ (2008) Resistance and resilience of a coral reef fish community to changes in coral cover. Mar Ecol Prog Ser 371:263-271

Ilarri MDI, de Souza AT, de Medeiros PR, Grempel RG, de Lucena Rosa IM (2008) Effects of tourist visitation and supplementary feeding on fish assemblage composition on a tropical reef in the Southwestern Atlantic. Neotrop Ichthyol 6:651-656

Jones GP, McCormick MI, Srinivasan M, Eagle JV (2004) Coral decline threatens fish biodiversity in marine reserves. PNAS 101:8251-8253

Kamukuru AT, Mgaya TD, Ohman MC (2004) Evaluating a marine protected area in a developing country: Mafia Islans Marine Park, Tanzania. Ocean Coast Manage 47:321-337

Kane CN, Brooks AJ, Holbrook SJ, Schmitt RJ (2009) The role of determining microhabitat and social organisation in determining the spatial distribution of a coral reef fish. Environ Biol Fish 83:1-10

Khalaf M, Crosby MP (2005) Assemblage structure of Butterflyfishes and their use as indicators of Gulf of Aqaba benthic habitat in Jordan. Aquatic Conserv: Mar Freshw Ecosyst 15:S27-S43

Kingsford MJ (2009) Contrasting patterns of reef utilisation and recruitment of coral trout (*Plectropomus leopardus*) and snapper (*Lutjanus carponotatus*) at One Tree Island, southern Great Barrier Reef. Coral Reefs 28:251-264

Kramer KL, Heck JrKL (2007) Top-down trophic shifts in Florida Keys patch reef marine protected areas. Mar Ecol Prog Ser 349:111-123

Kuffner IB, Brock JC, Grober-Dunsmore R, Bonito VE, Hickey TD, Wright CW (2007) Relationships between reef fish communities and remotely sensed rugosity measurements in Biscayne National Park, Florida, USA. Environ Biol Fish 78:71-82

Kulbicki M, Sarramegna S, Letourneur Y, Wantiez L, Galzin R, Mou-Tham G, Chauvet C, Thollot P (2007) Opening of an MPA to fishing: Natural variations in the structure of a coral reef fish assemblage obscure changes due to fishing. J Exp Mar Biol Ecol 353:145-163

Lecchini D, Tsuchiya M (2008) Spatial structure of coral reef fish communities at Kudaka Island (Ryukyu Archipelago), Japan. Ichthyol Res 55:321-327

Ledlie MH, Graham NAJ, Bythell JC, Wilson SK, Jennings S, Polunin NVC, Hardcastle J (2007) Phase shifts and the role of herbivory in the resilience of coral reefs. Coral Reefs 26:641-653

Lirman D (1999) Reef fish communities associated with *Acropora palmata*: relationships to benthic attributes. Bull Mar Sci 65:235-252

Loreto RM, Lara M, Schmitter-Soto JJ (2003) Coral reef fish assemblages at Banco Chinchorro, Mexican Caribbean. Bull Mar Sci 73:153-170

MacNeil MA, Graham NAJ, Polunin NVC, Kulbicki M, Galzin R, Harmelin-Vivien M, Rushton SP (2009) Hierarchical drivers of reef-fish meta-community structure. Ecology 90:252-264

Mateo I, Tobias WJ (2001) Distribution of shallow water coral reef fishes on the northeast coast of St. Croix, USVI. Caribb J Sci 37:210-226

McClanahan TR, Graham NAJ, Maina J, Chabanet P, Bruggemann JH, Polunin NVC (2008) Influence of instantaneous variation on estimates of coral reef fish populations and communities. Mar Ecol Prog Ser 340:221-234

McLain DK, Pratt AE (1999) Nestedness of coral reef fish across a set of fringing reefs. Oikos 53:53-67

Miller MW, Gerstner CL (2002) Reefs of an uninhabited Caribbean island: fishes, benthic habitat, and opportunities to discern reef fishery impact. Biol Conserv 106:37-44

Minte-Vera CV, de Moura RL, Francini-Filho RB (2008) Nested sampling: an improved visual-census technique for studying reef fish assemblages. Mar Ecol Prog Ser 367:283-293

Monaco ME, Friedlander AM, Caldow C, Christensen JD (2007) Characterising reef fish populations and habitats within and outside the US Virgin Islands Coral Reef National Monument: a lesson in marine protected area design. Fisheries Manag Ecol 14:33-40

Mumby PJ, Foster NL, Fahy EAG (2005) Patch dynamics of coral reef macroalgae under chronic and acute disturbance. Coral Reefs 24:681-692

Munday PL (2004) Habitat loss, resource specialisation, and extinction on coral reefs. Glob Change Biol 10:1642-1647

Nagelkerken I, Dorenbosch M, Verberk WCEP, de la Moriniere EC, van der Velde G (2000) Importance of shallow-water biotopes of a Caribbean bay for juvenile coral reef fishes: patterns in biotope association, community structure and spatial distribution. Mar Ecol Prog Ser 202:175-192

Nanami A, Nishihira M, Suzuki T, Yokochi H (2005) Species-specific habitat distribution of coral reef fish assemblages in relation to habitat characteristics in an Okinawan coral reef. Environ Biol Fish 72:55-65

Nardi K, Jones GP, Moran MJ, Cheng YW (2004) Contrasting effects of marine protected areas on the abundance of two exploited reef fishes at the sub-tropical Houtman Abrolhos Islands, Western Australia. Environ Conserv 31:160-168

Newman SJ, Williams DMcB (2001) Spatial and temporal variation in assemblages of Lutjanidae, Lethrinidae and associates fish species among mid-continental shelf reefs in the central Great Barrier Reef. Mar Freshwater Res 52:843-851

Ortiz DM, Tissot BN (2008) Ontogenetic patterns of habitat use by reef-fish in a Marine Protected Area network: a multi-scaled remote sensing and *in situ* approach. Mar Ecol Prog Ser 365:217-232

Patterson HM, Lindsay M, Swearer SE (2007) Use of sonar transects to improve efficiency and reduce potential bias in visual surveys of reef fishes. Environ Biol Fish 78:291-297

Pratchett MS, Wilson SK, Berumen ML, McCormick MI (2004) Sublethal effects of coral bleaching on an obligate coral feeding butterflyfish. Coral Reefs 23:352-356

Purkis SJ, Graham NAJ, Riegl BM (2008) Predictability of reef fish diversity and abundance using remote sensing data in Diego Garcia (Chago Archipelago). Coral Reefs 27:167-178

Russ GR (2003) Grazer biomass correlates more strongly with production than the biomass of algal turfs on a coral reef. Coral Reefs 22:63-67

Sabater MG, Carroll BP (2009) Trends in reef fish population and associated fishery after three millennia of resource utilisation and a century of socio-economic changes in American Samoa. Rev Fish Sci 17:318-335

Sandin SA, Smith JE, DeMartini EE, Dinsdale EA, Donner SD, Friedlander AM, Konotchick T, Malay M, Maragos JE, Obura D, Pantos O, Paulay G, Richie M, Rohwer F, Schroeder RE, Walsh S, Jackson JBC, Knowlton N Sala E (2008) Baselines and degradation of coral reefs in the northern Line Islands. PLoS ONE 3:1-11

Sano M (2000) Stability of reef fish assemblages: responses to coral recovery after catastrophic predation by *Acanthaster planci*. Mar Ecol Prog Ser 198:121-130

Schmitt EF, Sluka RD, Sullivan-Sealey KM (2002) Evaluating the use of roving diver and transect surveys to assess the coral reef fish assemblage off southeastern Hispaniola. Coral Reefs 21:216-223

Schroeder RE, Green AL, DeMartini EE, Kenyon JC (2008) Long-term effects of a ship-grounding on coral reef fish assemblages at Rose Atoll, American Samoa. Bull Mar Sci 82:345-364

Sheppard CRC, Spalding M, Bradshaw C, Wilson S (2002) Erosion vs. recovery of coral reefs after 1998 ElNino Chagos Reefs, Indian Ocean. Ambio 31:40-48

Shuman CS, Hodgson G, Ambrose RF (2005) Population impacts of collecting sea anemones and anemonefish for the marine aquarium trade in the Philippines. Coral Reefs 24:564-573

Syms C, Jones GP (2001) Soft corals exert no direct effects on coral reef fish assemblages. Oecologia 127:560-571

Thompson AA, Mapstone BD (2002) Intra-versus inter-annual variation in counts of reef fishes and interpretations of long-term monitoring studies. Mar Ecol Prog Ser 232:247-257

Tissot BN, Hallacher LE (2003) Effects of aquarium collectors on coral reef fishes in Kona, Hawaii. Conserv Biol 17:1759-1768

Tittensor DP, Micheli F, Nystrom M, Worm B (2007) Human impacts on the species-area relationship in reef fish assemblages. Ecol Lett 10:760-772

Tupper M, Rudd MA (2002) Species-specific impacts of a small marine reserve on reef fish production and fishing productivity in the Turk and Caicos Islands. Environ Conserv 29:484-492

Tuya F, Sanchez-Jerez P, Haroun RJ (2006) Populations of inshore serranids across the Canarian Archipelago: relationships with human pressure and implications for conservation. Biol Conserv 128:13-24

Unsworth RKF, Powell A, Hukom F, Smith DJ (2007) The ecology of Indo-Pacific grouper (Serranidae) species and the effects of a small scale no take area on grouper assemblage, abundance and size frequency distribution. Mar Biol 152:243-254

Van Nguyen L, Phan HK (2008) Distribution and factors influencing on structure of reef fish communities in Nha Trang Bay Marine Protected Area, South-Central Vietnam. Environ Biol Fish 82:309-324

Whitfield PE, Hare JA, David AW, Harter SL, Munoz RC, Addison CM (2007) Abundance estimates of the Indo-Pacific lionfish *Pterois volitans/miles* complex in the Western North Atlantic. Biol Invasions 9:53-64

Wielgus J, Ballantyne IV F, Sala E, Gerber LR (2007) Viability analysis of reef fish populations based on limited demographic informal. Conserv Biol 21:447-454

Williams ID, Walsh WJ, Tissot BN, Hallacher LE (2006) Impact of observers’ experience levels on counts of fishes in underwater visual surveys. Mar Ecol Prog Ser 310:185-191

Williamson DH, Russ GR, Ayling AM (2004) No-take marine reserves increase abundance and biomass of reef fish on inshore fringing reefs of the Great Barrier Reef. Environ Conserv 31:149-159

Wilson SK, Dolman AM, Cheal AJ, Emslie MJ, Pratchett MS, Sweatman HPA (2009) Maintenance of fish diversity on disturbed coral reefs. Coral Reefs 28:3-14

Wismer S, Hoey AS, Bellwood DR (2009) Cross-shelf benthic community structure on the Great Barrier Reef: relationships between macroalgal cover and herbivore biomass. 376:45-54

Yap HT (2009) Local changes in community diversity after coral transplantation. Mar Ecol Prog Ser 374:33-41
